# Ammonium chloride administration prior to exercise has muscle‐specific effects on mitochondrial and myofibrillar protein synthesis in rats

**DOI:** 10.14814/phy2.14797

**Published:** 2021-03-26

**Authors:** Amanda J. Genders, Evelyn C. Marin, Joseph J. Bass, Jujiao Kuang, Nicholas J. Saner, Ken Smith, Philip J. Atherton, David J. Bishop

**Affiliations:** ^1^ Institute for Health and Sport (iHeS) Victoria University Melbourne Victoria Australia; ^2^ Department of Medicine (Austin Health) The University of Melbourne Melbourne Victoria Australia; ^3^ MRC/ARUK Centre for Musculoskeletal Ageing Research Nottingham Biomedical Research Centre (BRC) National Institute for Health Research (NIHR) School of Medicine University of Nottingham Nottingham UK

**Keywords:** acidosis, exercise, mitochondria, protein synthesis, skeletal muscle

## Abstract

**Aim:**

Exercise is able to increase both muscle protein synthesis and mitochondrial biogenesis. However, acidosis, which can occur in pathological states as well as during high‐intensity exercise, can decrease mitochondrial function, whilst its impact on muscle protein synthesis is disputed. Thus, the aim of this study was to determine the effect of a mild physiological decrease in pH, by administration of ammonium chloride, on myofibrillar and mitochondrial protein synthesis, as well as associated molecular signaling events.

**Methods:**

Male Wistar rats were given either a placebo or ammonium chloride prior to a short interval training session. Rats were killed before exercise, immediately after exercise, or 3 h after exercise.

**Results:**

Myofibrillar (*p* = 0.036) fractional protein synthesis rates was increased immediately after exercise in the soleus muscle of the placebo group, but this effect was absent in the ammonium chloride group. However, in the gastrocnemius muscle NH_4_Cl increased myofibrillar (*p* = 0.044) and mitochondrial protein synthesis (0 h after exercise *p* = 0.01; 3 h after exercise *p* = 0.003). This was accompanied by some small differences in protein phosphorylation and mRNA expression.

**Conclusion:**

This study found ammonium chloride administration immediately prior to a single session of exercise in rats had differing effects on mitochondrial and myofibrillar protein synthesis rates in soleus (type I) and gastrocnemius (type II) muscle in rats.

## Graphical abstract

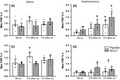

## 1 INTRODUCTION

Mitochondria have been associated with both exercise performance and health (Granata et al., 2016, 2018; Jacobs & Lundby, 2013; Jacobs et al., 2011; Nunnari & Suomalainen, 2012; Russell et al., 2014). Exercise training increases mitochondrial content and respiratory function (Granata et al., 2018), and this has been attributed to the cumulative effects of each exercise session on mitochondrial biogenesis (Granata et al., 2016; Holloszy, 1967; Hood, 2001; Little et al., 2011; Perry & Hawley, 2018; Russell et al., 2014) – which has been defined as “the making of new components of the mitochondrial reticulum” (Miller & Hamilton, 2012). This is a result of the coordinated expression of the nuclear and the mitochondrial genomes (Hood, 2001). However, not all changes in mRNA content translate to changes in protein content and the reliance on changes in mRNA to assess mitochondrial biogenesis has been criticised (Miller & Hamilton, 2012). Thus, in the current study, in addition to measuring the mRNA and protein content of key molecular signals associated with mitochondrial biogenesis, we have also measured mitochondrial protein synthesis (MitoPS) as it has been argued to be the best measure of mitochondrial biogenesis (Miller & Hamilton, 2012).

Exercise has also been reported to increase myofibrillar protein synthesis (MyoPS) (Bell et al., 2015; Di Donato et al., 2014). At the molecular level, MyoPS is regulated by the mammalian Target of Rapamycin (mTOR), which integrates nutritional and mechanical stimuli to generate an appropriate molecular response. Activation of the mTOR complex 1 (mTORC1) subsequently leads to the concurrent phosphorylation and activation of 70‐kDa ribosomal S6 protein kinase (p70S6K) and inhibition of 4E‐binding protein 1 (4EBP1), which in turn can lead to an increase in protein synthesis (Bodine et al., 2001). Again, while measures of mRNA and protein phosphorylation or content of this signaling pathway provide a snapshot of the likely changes induced by exercise, the measurement of MyoPS (as measured herein) is more indicative of the changes caused by exercise. A greater understanding of factors affecting exercise‐induced increases in MyoPS has a number of potential applications for those concerned with increasing or maintaining muscle mass in a variety of different scenarios (e.g., elderly or clinical populations).

Acidosis accompanies many clinical conditions, such as chronic renal failure and diabetic acidosis, which can be associated with loss of body protein (Caso et al., 2004). Intense muscle contractions, as seen with high‐intensity exercise, also lead to an accumulation of hydrogen ions (Bishop et al., 2007, 2010). This results in a decrease in muscle pH to values as low as 6.8 in the soleus and 6.6 in the EDL of rats (Bishop et al., 2010), with a similar decrease in the vastus lateralis of active women (Bishop et al., 2007). A decrease in pH is sufficient to decrease the capacity for lipid metabolism (Sahlin et al., 2008), oxidative phosphorylation (Jubrias et al., 2003), and to alter the expression and/or activity of some proteins involved in protein degradation and glucose metabolism (Bailey et al., 1996, 2006; Bento et al., 2005; Genders et al., 2019; Isozaki et al., 1996). We have previously shown that minimizing the decrease in intracellular pH during high‐intensity exercise training promotes greater improvements in mitochondrial respiration (Bishop et al., 2010). This leads to the hypothesis that intracellular pH may affect the cellular signaling pathways and genes that regulate mitochondrial biogenesis. In support, when humans ingest ammonium chloride (NH_4_Cl) the exercise‐induced mRNA expression of mitochondrial genes such as PGC‐1α and cytochrome c in skeletal muscle is decreased (Edge et al., 2015). However, it is unclear what the influence of these gene expression changes is on exercise‐induced mitochondrial protein synthesis. The effects of acidosis on myofibrillar protein synthesis are also controversial. Chronic renal failure patients (who have decreased extracellular pH) have increased protein synthesis (Garibotto et al., 1994), while decreasing extracellular and intracellular pH inhibited resting myofibrillar protein synthesis in rats in two studies (Balgi et al., 2011; Caso et al., 2004), but had no effect in another study (Maniar et al., 1994). However, there are no studies examining the effects of acidosis on exercise‐induced changes in protein synthesis. Therefore, although there is good evidence to suggest that the pH changes that occur with either high‐intensity exercise or administration of NH_4_Cl may alter signaling that initiates mitochondrial biogenesis, the overall response and influence is unclear.

The aim of this study was to determine the effect of mild physiological decreases in pH on MitoPS and MyoPS, and protein phosphorylation and mRNA expression of genes and proteins known to regulate these processes. The measurement of MitoPS was used as an indicative measure of mitochondrial biogenesis, while MyoPS was assessed to determine the production of new myofibrillar components. In addition, we also investigated the effects of NH_4_Cl ingestion on acute, exercise‐induced genes that are involved in the regulation of both mitochondrial biogenesis and myofibrillar protein synthesis in skeletal muscle. As there is evidence to suggest there is a differentiated response to exercise of metabolic signaling/effector proteins in human type I and II fibers (e.g., oxidative enzymes, AMPK signaling) (Christiansen et al., 2018; Henriksson & Reitman, 1976; Kristensen et al., 2015; MacInnis et al., 2017), including in response to pH (Bishop et al., 2010), we investigated these responses in both the soleus and gastrocnemius muscle. We hypothesized that short‐term changes in blood pH immediately prior to the commencement of an acute exercise session would decrease mitochondrial and myofibrillar protein synthesis, as well as the expression of activity‐induced genes and signaling proteins.

## 2 MATERIALS AND METHODS

### 2.1 Animals

This study was approved by the Victoria University Animal Ethics Committee (15/002). All procedures were performed according to the Australian Code of Practice for the Care and Use of Animals for Scientific Purposes (National Health and Medical Research Council, Australia, 8th Edition). Male Wistar rats were purchased from the Animal Resource Centre (Perth, Australia) at 6 weeks of age. Rats were housed 2–4 per cage in a temperature‐controlled room (18–22°C) and maintained with a chow diet (Specialty Feeds) and drink *ad libitum* on a 12:12 h light‐dark cycle. Treadmill exercise took place when the animals were 8 weeks old and 335 ± 34 g in mass. All exercise sessions took place in the daytime. Rats were acclimatized to the treadmill over 3 days using five separate sessions, 15 min in length. The first session consisted of 15 min on a non‐moving treadmill belt. The speed and incline of the treadmill was gradually increased until in the final acclimatization session it reached 0.25 m/s at a 10‐degree incline. An incremental exercise test was performed to assess the rats’ exercise capacity. The treadmill inclination was set at 10° and the test was started at a speed of 0.16 m/s, and then, the speed was increased 0.05 m/s every three minutes. Animals were removed from the treadmill when they could no longer keep up with the speed despite encouragement (air puff). The average top speed for the incremental exercise test was 0.48 ± 0.01 m/s. This was at slower speed than obtained in a previous study (0.62 m/s) (Bishop et al., 2010), which may have been due to the encouragement methods used in the two studies (air puff in the current study vs. electric shock in the previous study). At least 72 h after the test all animals were randomly given either a placebo (drinking water only) or 0.05 g/kg NH_4_Cl via gavage. Fifteen min after the gavage treatment animals performed a single high‐intensity exercise session at 80% of their top speed (approximately 0.38 m/s at a 10‐degree incline) for seven 2‐min intervals interspersed with 1 min of rest. Exercise commenced 15 min after oral gavage to allow blood pH to stabilize (Bishop et al., 2010). Prior to, immediately post, or 3 h after the completion of the exercise protocol, rats were humanely killed using 90 mg/kg i.p. pentobarbitone and the soleus and superficial white portion of the medial gastrocnemius was removed and immediately frozen in liquid nitrogen and stored at −80°C.

### 2.2 Measurement of mitochondrial protein synthesis (MitoPS) and Myofibrillar protein synthesis (MyoPS)

Researchers blinded to experimental group did analysis of samples for measurement of MitoPS and MyoPS. Rats were given 9.8 ml/kg deuterium oxide (D_2_O) (Sigma‐Aldrich) via oral gavage at the same time as they were given either the placebo or NH_4_Cl. Blood samples were collected from each animal upon sacrifice. To determine peak D_2_O body water enrichment, two animals received D_2_O before blood samples were collected from the tail vein after 10, 20, 40, 60, 120 and 180 min. Blood was collected in Lithium Heparin tubes, centrifuged and the plasma separated into aliquots and stored at −80°C. D_2_O enrichment of plasma was measured via acetone exchange (Yang et al., 1998). Briefly, 2 µl 10 N NaOH and 1 µl acetone was added to 100 µl of plasma before vortex mixing and incubation at room temperature for 24 h. Acetone was subsequently extracted into n‐heptane and injected into a gas chromatograph‐mass spectrophotometer (GC‐MS). D_2_O enrichment was measured via single ion monitoring (SIM) of m/z 58 and 59, referenced against a standard curve of known enrichment.

Isolation of myofibrillar and mitochondrial proteins was achieved as previously described (Wilkinson et al., 2013). Briefly, ~50 mg of muscle was homogenized with in ice‐cold homogenization buffer, and vortex mixed for 10 min, before centrifugation at 13,000 g for 5 min at 4°C, and supernatant removed. The resulting myofibrillar and mitochondrial containing pellet was further homogenized in ice‐cold mitochondrial extraction buffer in a dounce homogenizer, before centrifugation at 1,000 g for 5 min at 4°C. The mitochondria containing supernatant was then collected and pelleted by centrifugation at 13,000 g for 5 min at 4°C. The myofibrillar containing pellet was solubilized in 0.3 M NaOH at 37°C for 30 min, before centrifugation at 13,000 g for 5 min at 4°C, and myofibrillar supernatant removed and precipitated in 1 M PCA and centrifuged at 13,000 g for 5 min. Pelleted protein fractions were washed twice in 70% ethanol, before being hydrolyzed overnight at 110°C in 1 ml 0.1 M HCL and 1 ml H^+^ dowex resin. Hydrolyzed amino acids were eluted into 2 M NH4OH and evaporated to dryness. To determine deuterium labeling, protein‐bound alanine was converted to its tert‐butyldimethysilyl derivative and measured by SIM of m/z 260 and 261 by GC‐MS. The fractional synthetic rate (FSR) was calculated using the following equation:

$$\text{FSR}\left(\text{\textbackslash{}\% /h}\right)=\left[\left({\text{MPE}}_{\text{Ala}}\right)\right]/\left[3.7\times \left({\text{MPE}}_{\text{MW}}\right)\times t\right]\times 100$$

MPE_Ala_ represents protein bound alanine enrichment, MPE_MW_ represents plasma water enrichment (corrected for mean number of deuterium moieties incorporated per alanine, 3.7) and t signifies time in hours.

### 2.3 Western blotting

Muscle samples were homogenized in ice cold lysis buffer (0.05 M Tris pH 7.5, 1 mM EDTA, 2 mM EGTA, 10% glycerol, 1% Triton X‐100, 1 mM DTT) with Protease and Phosphatase Inhibitor cocktail (Cell Signaling Technologies). Lyzed samples were assayed for protein content and 5–10 µg protein was loaded onto TGX Stain‐Free FastCast Acrylamide gels. Proteins were separated by electrophoresis and then transferred onto PVDF membrane using the Trans‐Blot^®^ Turbo™ Blotting System (Bio‐Rad). Membranes were then blocked for 1 h at room temperature in TBST (TBS with 0.1% Tween 20 pH 7.6) with either 1% bovine serum albumin (BSA) or 5% skim milk powder. Membranes were then probed with the following primary antibodies overnight at 4°C at 1:1000 in TBST unless otherwise noted (all antibodies from Cell Signaling Technologies except where noted), phospho‐Thr180/Tyr182 p38 MAPK (#9211), total p38 MAPK (#9212), phospho‐CaMKII (#12716), total CaMKII (#3362), phosphoT172AMPK (#2531), total AMPK (#2532), phosphoSer2448mTOR (#5536), total mTOR (#2983), phosphoT389p70 S6 kinase (#9234), total p70 S6 kinase (#2708), HDAC5 (#20458), PGC‐1α (abcam #54481), p53 (absolute antibody), PPARα (#24509, abcam), Histone H3 (#24509), and LDHA (#2012). Blots were then washed with TBST prior to incubation with the appropriate HRP‐linked secondary antibody for 1 h at room temperature. Blots were developed using Clarity ECL (Bio‐Rad) and visualized using a ChemiDoc. All bands were quantified using ImageLab software (Bio‐Rad Laboratories). All phosphorylated and individual protein expression was normalized to total protein as determined from the stain‐free blot images.

### 2.4 Subcellular fractionation

Purified nuclear fractions were isolated from the soleus and gastrocnemius muscle using a combination of physical and chemical disruption methods as modified from (Dimauro et al., 2012). Approximately 50 mg muscle was homogenized in SEMH buffer (20 mM HEPES‐KOH pH 7.6; 220 mM mannitol; 70 mM sucrose, 1 mM EDTA; protease and phosphatase inhibitor cocktail) using a drill fitted Teflon pestle on ice. The homogenate was centrifuged at 500 g for 10 min at 4°C to separate the crude nuclear and cytosolic fractions. To further purify the nuclear fraction cell pellet was re‐suspended in SEMH buffer, vortexed, and centrifuged at 1,000 g for 15 min at 4°C. This step was then repeated before the pellet was re‐suspended in NET buffer (20 mM HEPES pH7.9, 1.5 mM MgCl2, 1.5 M NaCl, 0.2 mM EDTA, 20% glycerol, 1% triton X‐100 with protease and phosphatase inhibitor cocktail). The pellet was then vortexed for 15 s every 10 min for 30 min. This was followed by passing the solution through a 25G needle at least 10 times and two freeze thaw cycles. The solution was then centrifuged at 9,000 g for 30 min at 4°C, and the supernatant collected as the pure nuclear fraction. After assaying for protein content, all nuclear samples were precipitated with acetone to remove excess NaCl from the sample. Purity was assessed by western blotting for Histone H3 and LDHA (Figure 3i).

### 2.5 Quantitative RT‐PCR

RNA was extracted using TRIzol^®^ Reagent (Invitrogen, Thermo Fisher Scientific) as described in the manufacturer's instructions. Total RNA was extracted from approximately 10–20 mg of frozen muscle. Cellular membranes were disrupted using TissueLyzser II (Qiagen) for 2 × 2 min at 30 Hz with TRIzol^®^ Reagent. The homogenate was centrifuged (13,000 rpm for 15 min) and the RNA containing supernatant was removed. Homogenate was combined with chloroform (Sigma‐Aldrich) and RNA was then extracted using a standard protocol. The purity of each sample was assessed from the A260/A280 absorption ratio using a Nanodrop (Thermo). Total RNA concentration was also measured using the Nanodrop. RNA integrity of a subset of the samples was measured using a Bio‐Rad Experion microfluidic gel electrophoresis system (Bio‐Rad) and determined from the RNA quality indicator (RQI). All samples were of a good quality (RQI 9.3 ± 0.3) and protein contamination was low (A260/A280 1.96 ± 0.01). RNA was reverse transcribed to first strand cDNA from 1 µg of template RNA using a Thermocycler (Bio‐Rad) and Bio‐Rad iScript™ RT Supermix (Bio‐Rad) as per the manufacturer's instructions. Priming was performed at 25°C for 5 min and reverse transcription for 30 min at 42°C using random hexamers and oligo dTs. qPCR was performed with a QuantStudio 7 Flex (Applied Biosystems). Primers were either adapted from existing literature or designed using Primer‐BLAST (http://www.ncbi.nlm.nih.gov/tools/primer‐blast/) to include all splice variants, and were purchased from Sigma‐Aldrich. Primer specificity was confirmed from melting curve analysis. The PCR reaction (5 μl) contained 0.3 μM of each forward and reverse primer (Table 1) and 2.5 μl of SsoAdvanced Universal SYBR Green Supermix (Bio‐Rad). A serial dilution analysis was used to determine the amount of template cDNA. The standard thermocycling program consisted of a 95°C denaturation pre‐treatment for 10 min, followed by 40 cycles of 95°C for 15 s and 60°C for 60 s. Quantification of the target mRNA was normalized for differences in the amount of total RNA added to each reaction using reference mRNA (ACTB, cyclophilin and B2M). All samples were run in duplicate with template free controls, using an automated pipetting system (epMotion M5073, Eppendorf), and the mean Ct values for each sample were calculated. ΔCt was calculated as the difference between target and reference genes. RefFinder (Xie et al., 2012) was used to establish the stability of the reference genes. r18S was also trialled as a potential reference gene, but was found to be unsuitable.

**TABLE 1:** Primer sequences

Gene	Primer sequences	Accession number	Product size (bp)
PGC‐1α	F 5′‐ATA CAC AAC CGC AGT CGC AAC R 5′‐GCA GTT CCA GAG AGT TCC ACA C	NM_031347.1	148
COXIV	F 5′‐GCA GCA GTG GCA GAA TGT TG R 5′‐CGA AGG CAC ACC GAA GTA GA	NM_017202.1	79
Cytochrome c	F 5′‐TTC AAA AGT GTG CCC AGT GC R 5′‐TCC CCA GGT GAT ACC TTT GTT C	NM_012839.2	149
NRF1	F 5′‐CTACTCGTGTGGGACAGCAA R 5′‐AGCAGACTCCAGGTCTTCCA	NM_001100708.1	143
NRF2	F 5′‐TTT GGC AAG CCA AGA GCA AC R 5′‐TTG TTT CCT GTT CTG TTC CCC	NM_001108841.1	198
PDK4	F 5′‐GCA GTA GTC GAA GAT GCC TT R 5′‐ATG TGG ATT GGT TGG CCT GG	NM_053551.1	119
Tfam	F 5′‐AAT GTG GGG CGT GCT AAG AA R 5′‐ACA GAT AAG GCT GAC AGG CG	NM_031326.1	94
Reference genes			
ACTB	F 5′‐ACG ATA TCG CTG CGC TCG T R 5′‐GAC CCA TAC CCA CCA TCA CAC	NM_031347.1	138
B2M	F 5′‐ACC CAC CGA GAC CGA TGT A R 5′‐GGT CCC AGG TGA CGG TTT T	NM_012512.2	77
Cyclophilin	F 5′‐TCT GCA CTG CCA AGA CTG AG R 5′‐GTC CAC AGT CGG AGA TGG TG	NM_017101.1	147

Abbreviations: ACTB, beta‐actin; B2M, beta‐2microtubulin; F, forward; NRF, nuclear respiratory factor; PDK4, pyruvate dehydrogenase kinase 4; PGC‐1α, peroxisome proliferator‐activated receptor gamma coactivator 1‐alpha; R, reverse; Tfam, transcription factor A mitochondrial.

### 2.6 Statistical analysis

All results are expressed at mean ± *SD*. All protein and gene expression results were normalized to the No exercise placebo group. Data were analysed for statistical significance using one‐way ANOVA followed by Tukey or Games‐Howell post‐hoc test for pairwise comparisons (SPSS Statistics 22). Gene expression data were log transformed to improve the distribution prior to statistical analysis, as was protein expression data if not normally distributed. As myofibrillar protein synthesis from the gastrocnemius muscle was not normally distributed, an independent‐samples Kruskal–Wallis Test with Bonferroni correction for multiple comparisons was used to determine statistical significance. Significance was set at *p* < 0.05.

## 3 RESULTS

### 3.1 Myofibrillar and mitochondrial protein synthesis

**FIGURE 1:**
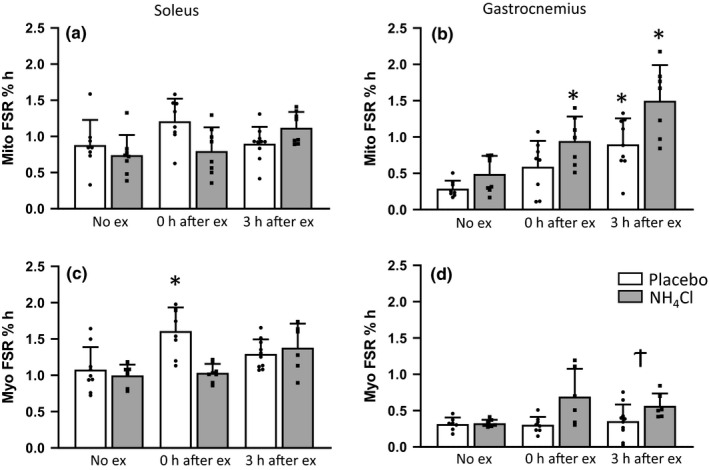
Myofibrillar and mitochondrial protein synthesis. Mitochondrial fractional synthesis rate (FSR) in the (a) soleus muscle (*n* = 8 for all groups, except 3 h ex placebo that is *n* = 10), and (b) in the gastrocnemius muscle (*n* = 8 for No ex placebo and NH_4_Cl and 0 h ex placebo, *n* = 7 for 0 h ex NH_4_Cl and 3 h ex NH_4_Cl, *n* = 9 for 3 h ex placebo). Myofibrillar FSR in the (c) soleus muscle (No ex pl *n* = 9, No ex NH_4_Cl *n* = 7, 0 h ex placebo and NH_4_Cl *n* = 8, 3 h ex placebo *n* = 10, 3 h ex NH_4_Cl *n* = 6), and (d) in the white gastrocnemius muscle (No ex placebo *n* = 7, No ex NH_4_Cl and 0 h ex placebo *n* = 8, 0 h ex NH_4_Cl and 3 h ex NH_4_Cl *n* = 6, 3 h ex placebo *n* = 10). Data are presented as mean ± *SD*, *p < 0.05 compared to No exercise placebo, † *p* < 0.05 compared to 3 h after exercise placebo

Myofibrillar and mitochondrial FSR was increased immediately after exercise in the soleus muscle of the placebo group; however, this effect was absent in the NH_4_Cl treated animals (Mitochondrial FSR No ex placebo 0.88 ± 0.35, 0 h after ex placebo 1.21 ± 0.31% h, *p* = 0.222; Myofibrillar FSR No ex placebo 1.08 ± 0.31, 0 h after ex placebo 1.61 ± 0.33% h, *p* = 0.036) (Figure 1a,c). In the gastrocnemius muscle, myofibrillar FSR tended to increase after exercise in animals administered NH_4_Cl, however; this was not statistically significant (No ex placebo 0.28 ± 0.13, 0 h after ex NH_4_Cl 0.69 ± 0.38; 3 h after ex NH_4_Cl 0.57 ± 0.29). However, there was a greater increase in myofibrillar FSR 3 h after exercise in the NH_4_Cl treated animals compared to the placebo treated (*p* = 0.044) (Figure 1d). Mitochondrial FSR in the gastrocnemius muscle was significantly increased in both groups 3 h after exercise, and in animals administered NH_4_Cl immediately after exercise (No ex placebo 0.29 ± 0.11; 0 h after ex NH_4_Cl 0.94 ± 0.33, *p* = 0.01; 3 h after ex placebo 0.90 ± 0.36, *p* = 0.028; 3 h after ex NH_4_Cl 1.50 ± 0.50, *p* = 0.003) (Figure 1b). Given these results, we next investigated protein signaling and mRNA expression of key proteins involved in mitochondrial biogenesis and protein synthesis.

### 3.2 Phosphorylation of signaling proteins

**FIGURE 2:**
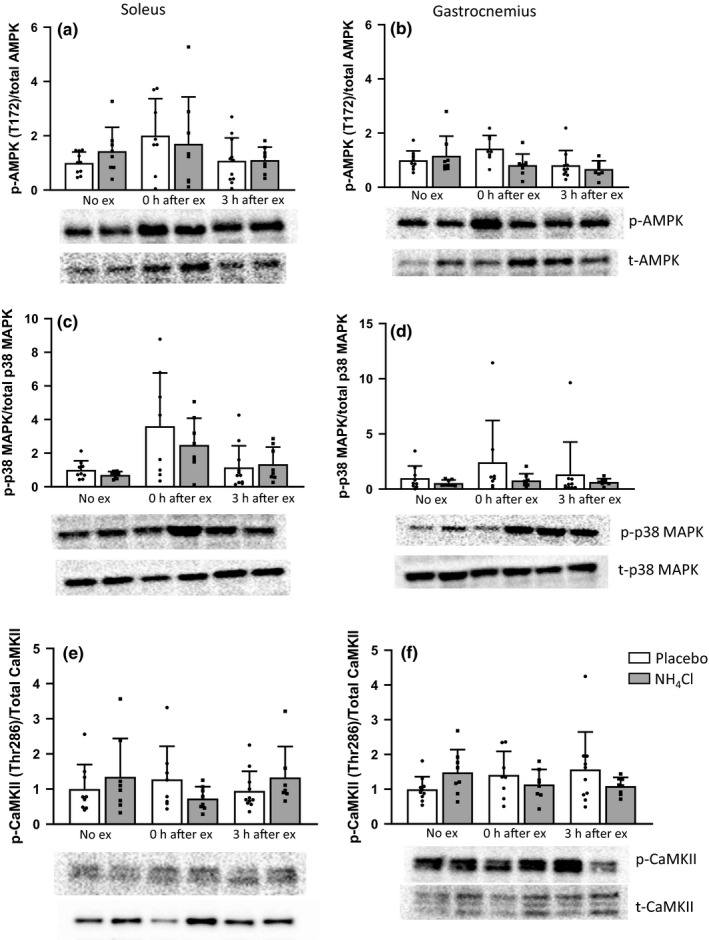
Protein phosphorylation of AMPK (a,b), p38 MAPK (c,d), and CaMKII (e,f) in soleus and white gastrocnemius muscle homogenates. Data are presented as mean ± *SD* relative to the No exercise placebo group. *p < 0.05 compared to the No exercise placebo group, Ϯ*p* < 0.05 compared to 0 h after ex placebo group (significant effect of NH_4_Cl administration). No ex placebo *n* = 10, 3 h ex placebo *n* = 11, all other groups *n* = 8

**FIGURE 3:**
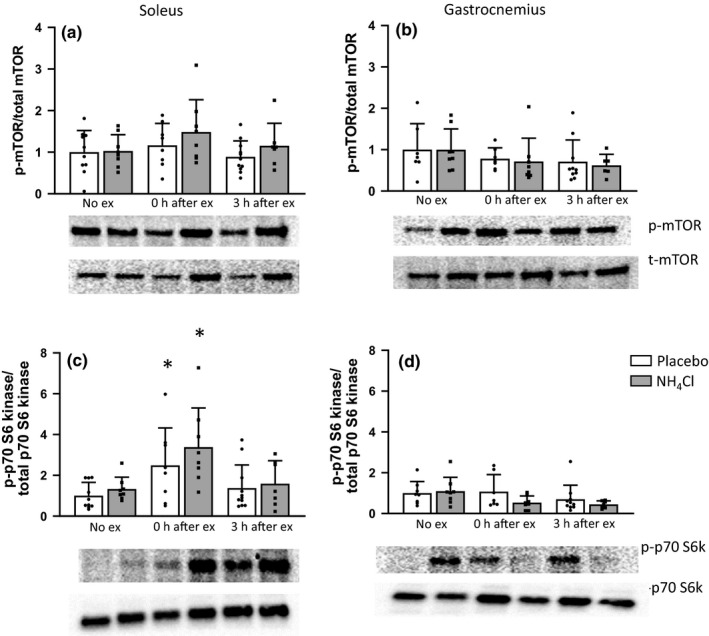
Protein phosphorylation of mTOR (a,b), and p70 S6 kinase (c,d) in the soleus and white gastrocnemius muscle homogenates. Data are presented as mean ± *SD* relative to the No exercise placebo group. *p < 0.05 compared to the No exercise placebo group, No ex placebo *n* = 10, 3 h ex placebo *n* = 11, all other groups *n* = 8

Neither high‐intensity exercise nor NH_4_Cl administration significantly influenced p‐AMPK content in the soleus muscle (Figure 2a). In the gastrocnemius muscle ANOVA analysis showed a significant difference between groups (*p* = 0.014); however, post‐hoc testing did not show any individual differences (Figure 2b). p‐p38 MAPK content in the soleus or gastrocnemius muscle were not significantly changed by exercise or NH_4_Cl administration (Figure 2c,d). Both p‐CaMKII (Figure 2e,f) and p‐mTOR (Ser2448) content (Figure 3a,b) was not changed in the soleus or gastrocnemius muscles. p‐p70 S6 kinase (T389) content was significantly increased immediately after exercise in the soleus muscle in both the placebo (2.5 ± 1.8, *p* = 0.02) and NH_4_Cl (3.4 ± 1.9, *p* = 0.0001) treatment groups (Figure 3c). p‐p70 S6 kinase content was decreased at the 0 h (0.5 ± 0.3) and 3 h (0.5 ± 0.02) time points in the gastrocnemius muscle of the NH_4_Cl treated animals; however, this was not statistically significant (Figure 3d).

**FIGURE 4:**
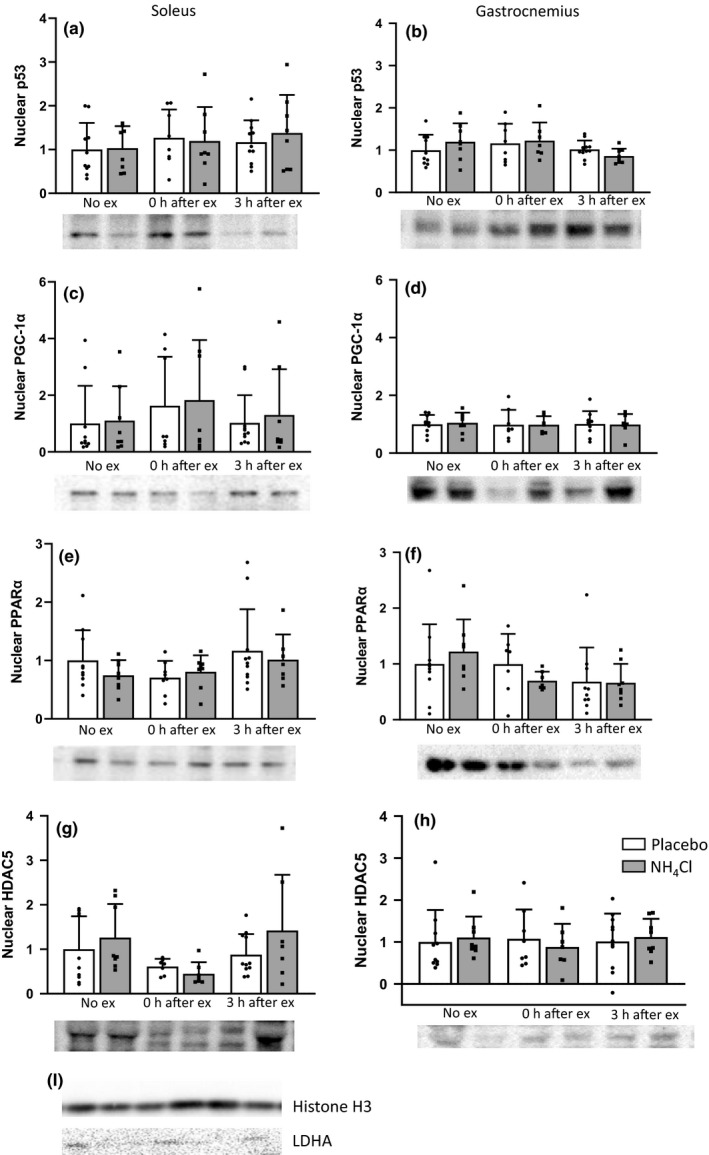
Nuclear content of p53 (a,b), PGC‐1α (c,d), PPARα (e,f) and HDAC5 (g,h) in the soleus and white gastrocnemius muscle. Representative blots of nuclear samples for Histone H3 and LDHA (i). Data are presented as mean ± *SD* relative to the No exercise placebo group. No ex placebo *n* = 10, 3 h ex placebo *n* = 11, all other groups *n* = 8

As these changes in protein phosphorylation did not explain the alterations seen in MitoPS and MyoPS, we investigated the nuclear localization of a number of proteins that influence transcription. However, neither exercise nor NH_4_Cl administration affected the quantity of nuclear p53, PGC‐1α, PPARα, or HDAC5 in the soleus or gastrocnemius muscle (Figure 4). Despite the absence of significant changes in these transcription factors, given the observed changes in MitoPS we next examined mRNA expression of key proteins involved in mitochondrial biogenesis.

### 3.3 mRNA expression

**FIGURE 5:**
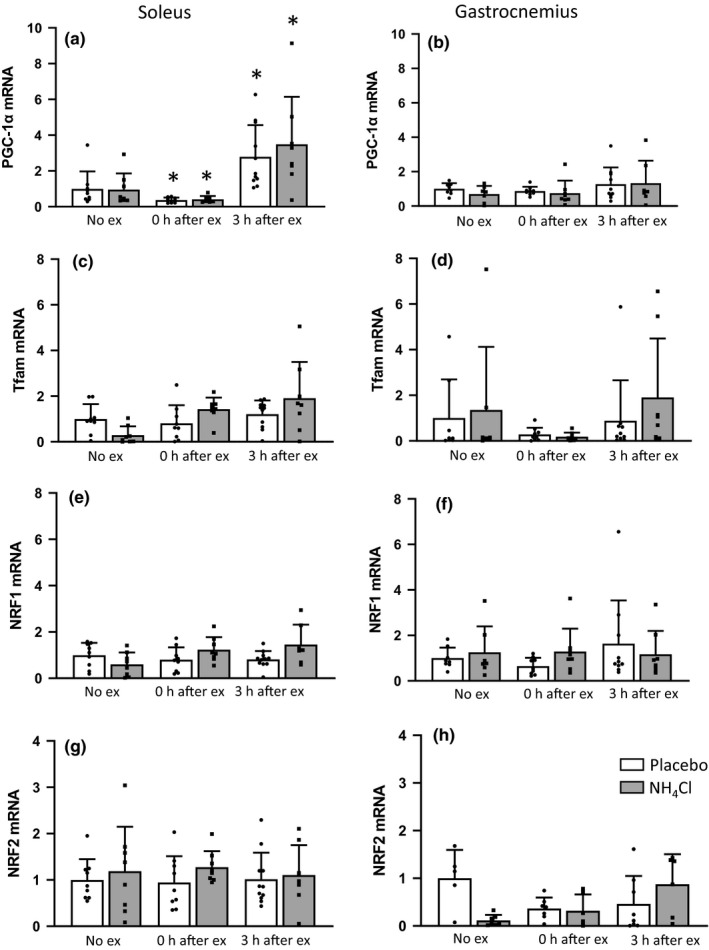
Gene expression in the soleus and gastrocnemius muscle. PGC‐1α (a,b), Tfam (c,d), NRF1 (e,f), NRF2 (g,h), Cytochrome c (i,j), COXIV (k,l), PDK4 (m,n) in the soleus and white gastrocnemius muscle. All data are presented as mean ± *SD* relative to the No exercise placebo group, *p < 0.05 compared to No exercise placebo, #*p* < 0.05 main effect of exercise. No ex placebo *n* = 10, 3 h ex placebo *n* = 11, all other groups *n* = 8

PGC‐1α mRNA expression increased approximately 300% 3 h after exercise (placebo 2.8 ± 1.8, *p* = 0.006; NH_4_Cl 3.5 ± 2.7, *p* = 0.006) in the soleus muscle (Figure 5a). PDK4 was increased approximately 30% 3 h after exercise (placebo 1.3 ± 0.7, NH_4_Cl 1.3 ± 0.7, main effect of exercise *p* ≤ 0.0001) (Figure 5m). These effects were not present in the gastrocnemius muscle (Figure 5b,n). No other gene expression changes were statistically significant.

## 4 DISCUSSION

This is the first study to investigate the effect of NH_4_Cl administration prior to high‐intensity interval training on protein synthesis in fractionated muscle (e.g., skeletal muscle mitochondrial and myofibrillar fractions). The current study found that NH_4_Cl administration immediately prior to a single session of exercise in male Wistar rats had differing effects on measures of mitochondrial and myofibrillar protein synthesis in the soleus and gastrocnemius muscle. In the soleus muscle, NH_4_Cl administration blunted the increases in MitoPS and MyoPS that were induced by exercise. However, in the gastrocnemius muscle NH_4_Cl administration, unexpectedly, increased post exercise MitoPS and MyoPS. The main findings of this study are presented in Table 2.

**TABLE 2:** Summary of post‐exercise results

	Soleus		Gastrocnemius	
	Placebo	NH_4_Cl	Placebo	NH_4_Cl
MitoPS	**–**	**–**	**↑**	**↑**
MyoPS	**↑**	**–**	**–**	**↑**
Phosphorylation				
p‐AMPK	**–**	**–**	**–**	**–**
p‐p38 MAPK	**–**	**–**	**–**	**–**
p‐CaMKII	**–**	**–**	**–**	**–**
p‐m‐TOR	**–**	**–**	**–**	**–**
p‐p70 S6K	**↑**	**↑**	**–**	**–**
mRNA content				
PGC‐1α	**↑**	**↑**	**–**	**–**
PDK4	**↑**	**↑**	**–**	**–**
NRF2	**–**	**–**	**–**	**–**
COXIV	**–**	**–**	**–**	**–**

Abbreviations: AMPK, AMP kinase; CaMKII, Ca2^+^/calmodulin‐dependent protein kinase II; COXIV, cytochrome c oxidase subunit 4; p38 MAPK, p38 mitogen‐activated protein kinase; mTOR, mechanistic target of rapamycin; NRF, nuclear respiratory factor; PDK4, pyruvate dehydrogenase kinase 4; PGC‐1α, peroxisome proliferator‐activated receptor gamma coactivator 1‐alpha; p70 S6K, ribosomal protein S6 kinase.

### 4.1 Exercise and MitoPS

In the current study, exercise increased MitoPS post exercise in both the soleus and the white gastrocnemius muscle of the placebo group. The results for MitoPS in the placebo group are similar to what has previously been reported. For example, 2 weeks of free access to cage wheel‐running increased MitoPS in the soleus muscle of rats (Holwerda et al., 2020). The increase in MitoPS in the white gastrocnemius for the placebo group is also in line with previous work in mice, in which no change in MitoPS was observed immediately post exercise but an increase in MitoPS was apparent at 3 and 6 h post exercise (Philp et al., 2015).

A novel finding of the present study was that these same increases in MitoPS were not apparent in the soleus muscle of the NH_4_Cl group. We are not aware of any previous research that has investigated the effect of pre‐exercise NH_4_Cl ingestion on exercise‐induced protein synthesis. However, these results are consistent with the greater training‐induced changes in mitochondrial respiration in the soleus muscle when rats ingested sodium bicarbonate (NaHCO_3_) prior to exercise training, which reduced hydrogen ion accumulation during exercise (Bishop et al., 2010). This same effect was not observed in the gastrocnemius in the present study, and there was an increase in MitoPS post exercise in both the placebo and NH_4_Cl groups. Differences in mitochondrial content and muscle recruitment during exercise may explain the different responses to NH_4_Cl in the soleus and gastrocnemius muscles. In addition, differences in muscle fiber type and blood flow (Armstrong & Laughlin, 1985; Armstrong & Phelps, 1984) between the soleus and white gastrocnemius muscle (higher blood flow in resting type I fibers, but a greater response to exercise in type II fibers), as well as muscle buffering capacity (higher buffering capacity in type II fibers compared to type I fibers) (Dolan et al., 2019; Weston et al., 1996), may help to explain the different response of the two muscles. Differences in blood flow are likely to influence hydrogen ion delivery to the muscle, whilst different fiber types have differing expression of proteins involved in muscle pH buffering, and this may influence the ability of the muscles to respond to changes in blood pH.

### 4.2 Exercise and MyoPS

An increase in MyoPS in the soleus muscle was detected immediately post exercise in the placebo group (but had returned to baseline levels at 3 h). Consistent with our findings, endurance exercise has been shown to increase MyoPS in the soleus muscle of rats with access to a running wheel (Holwerda et al., 2020). However, the MyoPS results of the gastrocnemius for the placebo group are in contrast with a previous study that demonstrated an increase in MyoPS (at 0.5, 3, and 6 h post exercise) in the gastrocnemius muscle of mice following endurance exercise (Philp et al., 2015). Our exercise protocol (7 × 2‐min intervals at 0.38 m/s, 10° incline) was slightly more intense, but much shorter in duration than the protocol adopted by Philp and colleagues (Philp et al., 2015) (1 h at 0.30 m/s, 5° incline). The considerably shorter duration and higher intensity of our exercise protocol may have contributed to the differences in gastrocnemius MyoPS observed between the studies (Bell et al., 2015; Di Donato et al., 2014).

Another novel finding of the present study was that there was no after exercise increase in MyoPS in the soleus for the NH_4_Cl group. It is difficult to make a direct comparison with previous studies as no study has investigated the effect of pre‐exercise acid ingestion on exercise‐induced protein synthesis in different muscles. However, a previous study had reported that NH_4_Cl ingestion had an inhibitory effect on resting mixed‐muscle protein synthesis (and increased protein degradation) in the soleus muscle in rats, 24 h following administration (Caso et al., 2004). This finding supports our post‐exercise results, although we did not see any difference in MyoPS between the placebo and NH_4_Cl treated animals who did not exercise.

In contrast, in the gastrocnemius muscle there was no increase in MyoPS in the placebo group but an increase in the NH_4_Cl group at 3 h. Although a previous study reported that NH_4_Cl ingestion decreased resting mixed‐muscle protein synthesis in the gastrocnemius of rats (Caso et al., 2004), the inhibitory effect of acidosis on muscle protein synthesis is not always seen. One previous study reported no effect on protein synthesis in the gastrocnemius muscle in rats that had developed acidosis (Maniar et al., 1994). Furthermore, in patients with chronic renal failure (who exhibit metabolic acidosis), muscle protein synthesis rates (in the forearm) were reported to be increased compared to a healthy control group (Garibotto et al., 1994). While this increase was offset by significantly increased protein degradation, it is consistent with the post‐exercise increase in MyoPS for the NH_4_Cl group in the gastrocnemius muscle in the present study. However, it should be noted that the duration of acidosis in this study was of a short duration compared to the chronic acidosis seen in clinical populations. Although technically challenging, assessment of muscle protein degradation with NH_4_Cl administration would help to further explain the full influence of the decreases in pH on myofibrillar protein synthesis (Tipton et al., 2018). This may also help elucidate the mechanisms involved in clinical conditions such as renal disease or diabetic ketosis where patients often have a loss of body protein (Caso et al., 2004), as well as worse exercise tolerance and larger increases in blood pressure with exercise (Sprick et al., 2019).

### 4.3 Cell signaling responses

Given the effects of NH_4_Cl administration on MitoPS, we also investigated some key cell signaling events associated with mitochondrial biogenesis. Despite their known roles in the initiation of mitochondrial biogenesis (Bergeron et al., 2001; Brandauer et al., 2015; Coffey & Hawley, 2007; Drake et al., 2016), there were few clear effects of NH_4_Cl administration on the phosphorylation of AMPK, p38 MAPK, or CaMKII. The absence of any changes in AMPK phosphorylation in the soleus muscle is consistent with our previous results in cultured L6 myocytes, where we found no significant change in AMPK phosphorylation with a more acidic pH (Genders et al., 2019). NH_4_Cl administration prior to exercise also did not result in any changes in the nuclear localization of key proteins, or any clear changes in the expression of genes, associated with mitochondrial biogenesis.

We also investigated the activation of proteins within the Akt‐mTOR‐p70S6K molecular signaling pathway, which have been reported to regulate exercise‐induced protein synthesis (Bodine et al., 2001; Drummond et al., 2009). MTORC1 is a critical regulator of translation initiation and ribosome biogenesis and plays an important role in cell growth control (Manning & Cantley, 2007). In the current study, however, there was no change in p‐mTOR^ser2448^ post exercise in either the soleus or gastrocnemius muscle for both groups. There were however, increases in p‐p70S6K^T389^ in the soleus muscle of both groups at 0 h, and there was a decrease in phosphorylation of p70S6K at 0 and 3 h post exercise in the gastrocnemius for the NH_4_Cl group. While increases in protein synthesis sometimes correspond with changes in the mTOR signaling pathway (Brook et al., 2015), this is not always the case (Atherton et al., 2010; Philp et al., 2015; Saner et al., 2020). Furthermore, a study reported an increase in both MyoPS and MitoPS in the gastrocnemius of mice in the hours following endurance exercise, which occurred without an increase in p‐mTOR (Philp et al., 2015). This same study reported decreases in p‐p70S6K at 6 h post‐exercise in the gastrocnemius muscle, which also occurred in the NH_4_Cl group in this study. Differences in mTOR‐related signaling between the current study and previous reports may be a result of differences in exercise protocols, with mTOR signaling as well as Myo and Mito PS having previously been shown to be dependent on exercise intensity and type (Di Donato et al., 2014; Philp et al., 2015). In addition, the transient nature of mTOR signaling (Di Donato et al., 2014) and the timing of when our samples were collected may explain the absence of mTOR phosphorylation and signaling in the presence of changes in both MyoPS and Mito PS. It is also possible that phosphorylation of mTOR at Ser2448 is either not crucial for regulation of mTOR activity or that the type of exercise used in this study is able to stimulate Myo/Mito PS independently of mTORC1 activation (Atherton et al., 2010; Philp et al., 2015).

We saw smaller or less consistent effects of both a single exercise session and NH_4_Cl administration on mRNA content and protein phosphorylation in the white gastrocnemius muscle in comparison to the soleus muscle, with a few exceptions. This was most likely due to the exercise intensity used in this study. While muscles comprised predominately of slow oxidative fibers, such as the soleus muscle, are recruited at relatively low exercise intensities, muscles such as the white part of the gastrocnemius are comprised of mainly fast glycolytic fibers and are recruited at much higher running speeds (approximately 25 m/min) (Armstrong & Laughlin, 1985; Armstrong & Phelps, 1984). In the current study the rats ran at approximately 0.38 m/s (i.e. approximately 23 m/min) and this may have contributed to the smaller and less consistent exercise effects in the white gastrocnemius muscle. It may also have been necessary to use a higher exercise intensity to see larger or more consistent effects on protein signaling and mRNA content. In support of this, two genes that can be dramatically increased with high‐intensity exercise (PGC‐1α and PDK4) (Granata et al., 2017; Pilegaard et al., 2003) were only moderately increased in this study (although still statistically significant). Unfortunately, we were unable to measure muscle pH and lactate in the soleus and white gastrocnemius or blood pH. However, we do know from previous experiments in rats and humans that small changes in blood pH are able to elicit a biological response, and occur with similar doses of NH_4_Cl (Bento et al., 2005; Bishop et al., 2010; Caso et al., 2004; Edge et al., 2015). For example, our dosage of 0.05 g/kg body weight was given using NH_4_Cl dissolved in the volume of D_2_O required for the protein synthesis measurement, made up as a concentration of 95 mM. This compares well to Caso et al. (2004) who used two doses of 20 Mm/kg over 24 h, and Bento et al. (2005) who used 250 mM NH_4_Cl in the rats’ drinking water. Both of these studies saw a pH drop of approximately 0.2 pH units.

In summary, these results show that NH_4_Cl administration prior to exercise has a muscle‐specific influence on exercise‐induced MitoPS and MyoPS. The effects were in contrast with our original hypothesis, which was informed by previous studies that showed exercise‐induced protein synthesis would decrease in both the soleus and gastrocnemius muscle (Balgi et al., 2011; Caso et al., 2004; Edge et al., 2015). One unique aspect of our study was the ability to look at the response in two different muscles with differing fiber type compositions. The results of this study suggest that the response to a drop in pH may be dependent on the fiber‐type composition within these muscles, as well as their differing recruitment during exercise. However, these fiber‐specific responses may also be influenced by differing blood flow and mitochondrial content (which are both higher in oxidative fibers) (Armstrong & Laughlin, 1985; Armstrong & Phelps, 1984). The results may have implications for populations who experience a greater decrease in pH during physical activity (e.g. those with diabetes and chronic kidney disease) (Beetham et al., 2019; Caso et al., 2004; Regensteiner et al., 1998), however, warrant further investigation over a longer period.

## CONFLICT OF INTEREST

The authors have no conflicts of interest to declare.

## AUTHOR CONTRIBUTIONS

A.J.G, K.S., P.J.A., and D.J.B. designed the research; A.J.G., E.C.M., J.J.B., J.K., and N.J.S. performed the research. A.J.G., N.J.S, and D.J.B. drafted the manuscript. All authors contributed to and approved the final version of this manuscript.

## Data Availability

The data that support the findings of this study are available from the corresponding author upon reasonable request.
